# Understanding the functional role of membrane confinements in TNF-mediated signaling by multiscale simulations

**DOI:** 10.1038/s42003-022-03179-1

**Published:** 2022-03-11

**Authors:** Zhaoqian Su, Kalyani Dhusia, Yinghao Wu

**Affiliations:** Department of Systems and Computational Biology, Albert Einstein College of Medicine, 1300 Morris Park Avenue, Bronx, NY 10461 USA

**Keywords:** Computational biophysics, Extracellular signalling molecules

## Abstract

The interaction between TNFα and TNFR1 is essential in maintaining tissue development and immune responses. While TNFR1 is a cell surface receptor, TNFα exists in both soluble and membrane-bound forms. Interestingly, it was found that the activation of TNFR1-mediated signaling pathways is preferentially through the soluble form of TNFα, which can also induce the clustering of TNFR1 on plasma membrane of living cells. We developed a multiscale simulation framework to compare receptor clustering induced by soluble and membrane-bound ligands. Comparing with the freely diffusive soluble ligands, we hypothesize that the conformational dynamics of membrane-bound ligands are restricted, which affects the clustering of ligand-receptor complexes at cell-cell interfaces. Our simulation revealed that only small clusters can form if TNFα is bound on cell surface. In contrast, the clustering triggered by soluble TNFα is more dynamic, and the size of clusters is statistically larger. We therefore demonstrated the impact of membrane-bound ligand on dynamics of receptor clustering. Moreover, considering that larger TNFα-TNFR1 clusters is more likely to provide spatial platform for downstream signaling pathway, our studies offer new mechanistic insights about why the activation of TNFR1-mediated signaling pathways is not preferred by membrane-bound form of TNFα.

## Abstract (web summary)

Su et. al. provide mechanistic insights and demonstrate the impact of membrane-bound TNFα in the clustering of TNFR1 receptor clustering.

## Introduction

Proteins in the tumor necrosis factor (TNF) superfamily function as indispensable ligands to trigger signaling pathways that are involved in not only the maintenance of tissue homeostasis and development, but also the regulation of the immune system^1–3^. They are type II transmembrane (TM) proteins which are characterized by the trimeric structure with three-fold symmetry through their C-terminal TNF homology domain (THD)^4–6^. All ligands in the superfamily are initially presented on cell surfaces, but most of them also occur as soluble variants after their “stalk” regions (the linker between the extracellular THD and transmembrane domains) are cleaved by metalloproteases^6^. With only a very few exceptions, the TNF superfamily members form interactions with their targets which belong to the TNF receptor (TNFR) superfamily^7^. The trimeric ligands can simultaneously bind to three receptors, leading into the formation of a ligand-receptor complex with 3:3 stoichiometry^8^. It has been observed in various systems of TNFR superfamily that the intracellular signaling pathways can only be effectively activated after these TNF-TNFR complexes are further assembled into higher-order clusters^9–13^. It was also found that receptors in some cases can even form pre-assembly on cell surface prior to ligand binding^12,14^.

Among the multifaceted mechanisms that mediate receptor clustering, the most common one is the lateral interactions between receptors, so called the “*cis*-interaction”, through their preligand binding assembly domain (PLAD)^14,15^. These PLAD regions are not only functionally conserved across the TNFR superfamily, but also do not spatially interfere with the ligand-receptor interaction, denoted as the “*trans*-interaction” hereafter. While some receptors in the TNFR superfamily can be robustly activated by soluble ligands, others can only be activated by ligands that are in the membrane-bound state^16^. More interestingly, evidences show that for those receptors that failed to be activated by soluble ligands, their activation can be rescued by anchoring the ligands to the cell surfaces^17,18^. This indicates that the cellular environments and membrane confinement of TNF ligands play an essential role in modulating the receptor clustering. However, it is not fully understood what the underlying mechanism of this observation is.

Intuitively, the confinement of TNF ligands on the surface of the plasma membrane leads to their loss of both translational and rotational degrees of freedom^19,20^. Comparing with the freely diffusive soluble ligands, we hypothesize that the conformational dynamics of membrane-bound ligands are changed by these constraints which further affects the kinetics of ligand-receptor interactions and clustering at cell-cell interfaces. Similarly, we hypothesize that preassembly of TNF receptors on the surface of the plasma membrane could also provide additional restrain to receptor’s conformational dynamics, thus leading to higher affinities for their ligands. Here, we use computational simulations to test these hypotheses. Computational modeling holds advantages to test variable conditions of a biomolecular system with the mechanistic details that are unapproachable by current experimental methods, not mentioning that measurements of receptor clustering in living cells with high spatial-temporal resolutions have only been successfully achieved in a limited number of cases. A large variety of computational models have recently been utilized to study the dynamics and functions of proteins in TNF or TNFR superfamily^21–24^. Unfortunately, simulation approaches relying on high-resolution structural details of individual proteins, such as molecular dynamics (MD) simulation, have difficulty reaching the timescale of ligand-receptor clustering^25–30^. In contrast, simulation approaches on the lower resolution, such as partial differentiation equations (PDE) and agent-based modeling (ABM), aimed to describe how collective behaviors of membrane receptors lead to spatial patterning on the subcellular level^31–40^. However, molecular details of receptors are rarely incorporated in these methods. One possibility to compensate the limitations of computational methods on different levels is the development of multiscale modeling technique^41–45^.

**Fig. 1:**
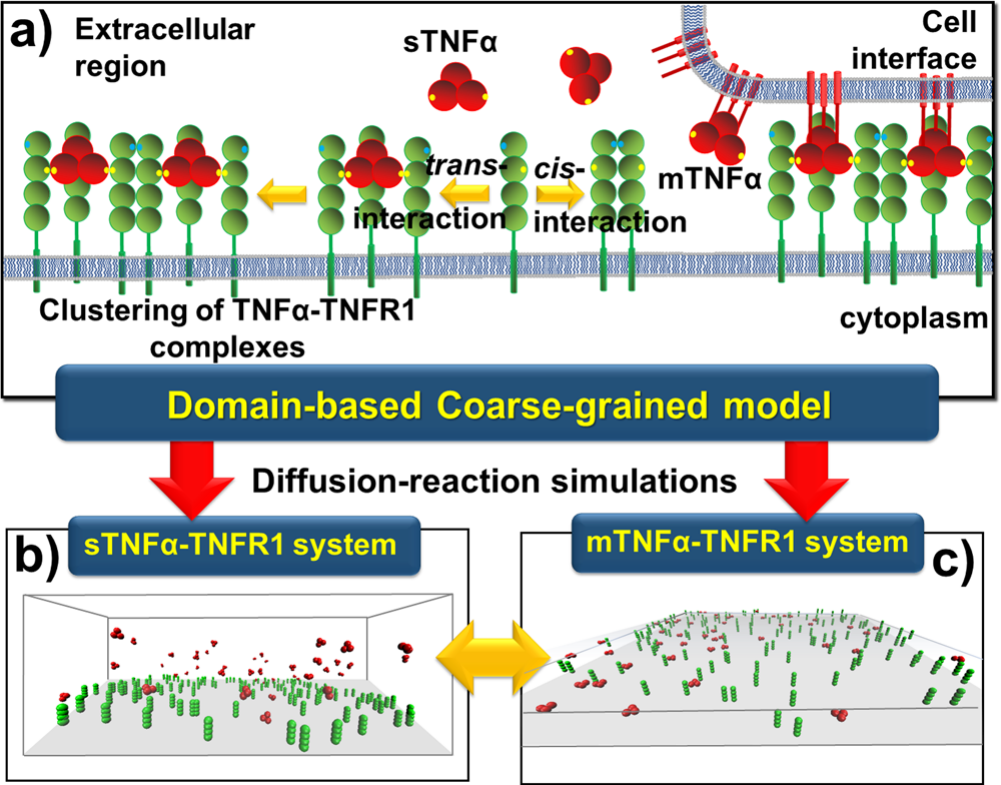
The multiscale simulation framework. TNFα ligands (red) are initially presented on cell surfaces (mTNFα). They also occur as soluble variants (sTNFα) after their “stalk” regions are cleaved by metalloproteases (**a**). The trimeric ligands can simultaneously form “*trans*-interactions” with three receptors TNFR1 (green). Additionally, two TNFR1 receptors can also form a “*cis*-interaction”, through their PLAD regions (green dots) which do not spatially interfere with the *trans*-binding sites (yellow dots). It was found that the activation of TNFR1-mediated signaling pathways is more preferred by sTNFα than mTNFα, while both forms of ligands can further induce the aggregation of TNFR1 into nanoscale clusters through the combination of *trans*- and *cis*-interaction. Using a domain-based coarse-grained model and diffusion-reaction simulation algorithm, we compare the system in which clustering of TNFR1 is induced by soluble TNFα (**b**) to the system in which clustering is induced by membrane-bound TNFα (**c**).

Given this background, a multiscale simulation framework is constructed in this study by integrating all-atom MD simulations with a domain-based coarse-grained (CG) diffusion-reaction model. It was found that the activation of TNFR1-mediated signaling pathways is preferentially through the soluble form of TNFα instead of the membrane-bound form^46,47^. Therefore, we applied the multiscale framework to the interaction between ligand TNFα and its receptor TNFR1 as a test model (Fig. 1). We compare the system in which clustering of TNFR1 is induced by soluble TNFα (sTNFα) to the system in which clustering is induced by membrane-bound TNFα (mTNFα). We found that the size of clusters formed by complexes between TNFR1 and sTNFα is statistically larger than the clusters formed by complexes between TNFR1 and mTNFα. This result provides the insight of why soluble ligand is more likely to activate TNFR1. Our study demonstrated that the environment of cell membrane can play an important role to regulate the spatial pattern of ligand-receptor clustering in situ.

## Results and discussions

**Fig. 2:**
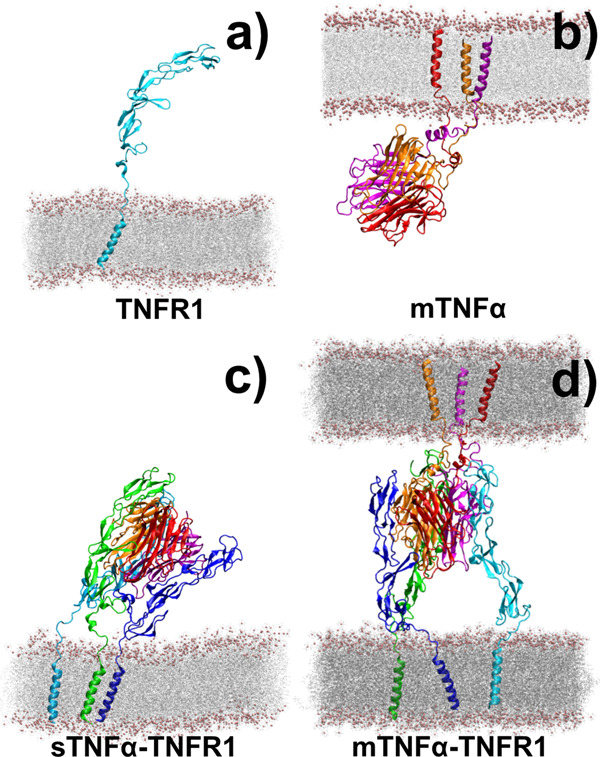
The all-atom structural models of four specific cellular systems. The conformational dynamics of ligand and receptor in these systems was analyzed by molecular dynamics simulations. The first system is the monomeric receptor TNFR1 on plasma membrane (**a**). The second system is the membrane-anchored TNFα ligand trimer (**b**). The third system is the complex formed between soluble ligand trimer and three membrane-bound TNFR1 receptors (sTNFα-TNFR1) (**c**). Finally, the last system is the complex formed between three membrane-bound TNFR1 receptors and a trimeric ligand TNFα which transmembrane regions are attached to an opposite plasma membrane (mTNFα-TNFR1) (**d**).

In order to estimate how membrane confinement of TNF ligand can affect its association with TNFR receptor, and distinguish the *cis*-interaction induced by membrane-bound ligands from the *cis*-interaction induced by soluble ligands, all-atom molecular dynamics simulation was applied to study the conformational fluctuations in five systems. The first one is the monomeric receptor on plasma membrane (TNFR1 as shown in Fig. 2a); the second one is the membrane-anchored ligand trimer (mTNFα as shown in Fig. 2b); the third one is the complex formed between soluble ligand trimer and three membrane-bound receptors (sTNFα-TNFR1 as shown in Fig. 2c); and the fourth one is the complex formed between three membrane-bound receptors and a trimeric ligand attached to the opposite plasma membrane (mTNFα-TNFR1 as shown in Fig. 2d). Finally, the last system is a *cis*-dimer formed by two TNFR1 on the plasma membrane (shown in Fig. S1).

**Fig. 3:**
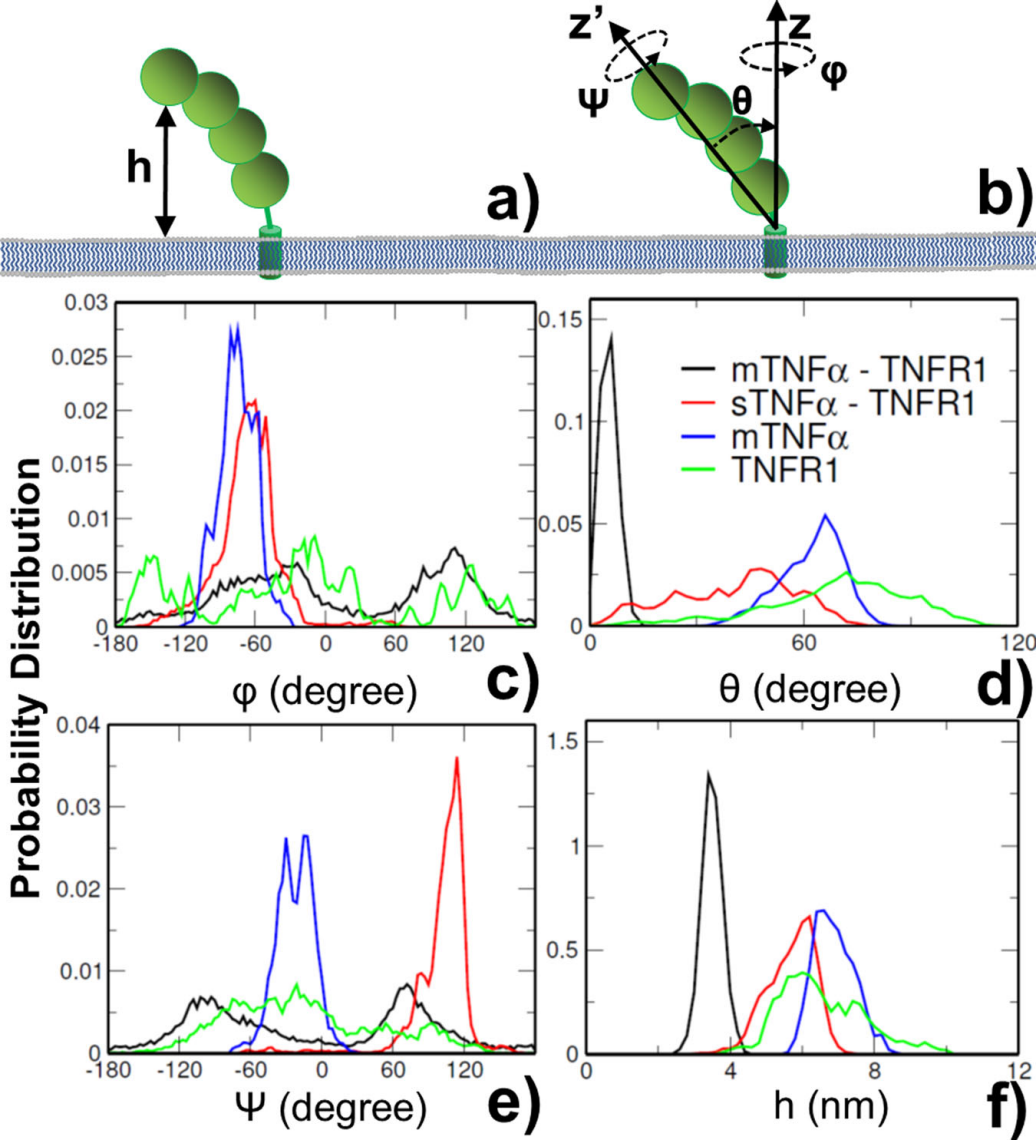
The distributions of conformational parameters derived from molecular dynamic simulations. These parameters include: the translational fluctuations of proteins along membrane normal, h, as shown in **a**, and three Euler angles which characterize the rotational phase space, as illustrated in **b**. The distributions of the angle around the long principal axis z′ of the protein ψ are shown in **c** as indexed by curves with different colors. the distributions of the tilting angle between this principal axis and the membrane normal θ are shown in **d**; and the distribution of the angle around the membrane normal z φ are shown in **e**. Similarly, detailed distributions of translational fluctuations are shown in **f** for proteins in four modeled systems.

We analyzed the conformational dynamics of ligand, receptor, and their complex formed under different conditions by MD simulations. In detail, the values of all conformational parameters were estimated from the five simulation systems. In addition to the range of translational fluctuations Δh for a corresponding membrane-bound molecule along its membrane normal (Fig. 3a), the range of volume in the rotational phase space of the molecule can be further characterized by three Euler angles as ∆ω = Δψ × Δφ (1 − cosΔθ), in which ψ is defined as the angle around the long principal axis z′ of the protein, θ is the tilting angle between this principal axis and the membrane normal, and φ is defined as the angle around the membrane normal z, as shown in Fig. 3b. The distributions of all these parameters were derived for each system based on the molecular conformations generated along the corresponding trajectory. The range of each parameter was then simply approximated as twice the standard deviations of its distributions. We assume this simplified calculation can capture the basic dynamic features of the ligand-receptor systems, although a thorough estimation of configurational volumes should involve more rigorous but computationally more expensive method such as thermodynamic integration, which is beyond the scope of current study.

The specific distributions of conformational fluctuations along both translational and rotational degrees of freedom are plotted from Fig. 3c–f for the four simulation systems illustrated in Fig. 2. Detailed ranges of all conformational parameters were calculated from these distributions and can be found in the supporting information as Table S2. The figures show that the profiles of distributions in different systems are highly distinguishable. For instance, comparing with other three systems, the simulation of membrane-bound TNFR1 monomer forms wide distribution along all four conformational degrees of freedom, as shown by green curves in the figures. In contrast, the distributions of conformational angles ψ and φ become much narrower when TNFR1 receptors form complex with soluble TNFα, as shown by the red curves in Fig. 3c, e respectively. This can be explained by the fact that all three receptors in the complex are tethered to the membrane (Fig. 2c), leading into multiple degrees of constraints to conformational fluctuations.

Similarly, the dynamics of trimeric ligand TNFα shows narrow distributions along conformational angles ψ and φ, as shown by the blue curves in Fig. 3c, e respectively. This is also due to the fact that all three subunits of the ligand are tethered to the membrane (Fig. 2b), thus causing higher level of confinements. Finally, additional restrictions to the conformational dynamics were introduced after both ligand and receptors are anchored to membrane in the mTNFα-TNFR1 complex (Fig. 2d). This is confirmed by the much narrower distributions along the tilting angle θ and the translational fluctuations h were obtained, as shown by the black curves in Fig. 3d, f respectively. Another feature is that the entire ligand-receptor complex sits more upright at the interface of two plasma membranes, which is indicated by the small tilting angle (black curve in Fig. 3d). As a result, the complex can rotate along the membrane normal more easily, leading into the wide distribution of conformational angle φ (black curve in Fig. 3c). Moreover, due to the small tilting angle, the long principal axis z′ of the complex is almost aligned to the membrane normal, leading into the similar profiles in the distributions between conformational angles ψ and φ (black curves in Fig. 3c, e). Taken together, the simulation results illustrate that the conformational dynamics of ligand, receptor, and their complex are closely regulated by different degrees of membrane confinements.

Additionally, we also compared the conformational fluctuations of TNFR1 in the monomeric state with the conformational fluctuations when it is in the *cis*-dimer. The specific distributions along both translational and rotational degrees of freedom are plotted in Fig. S2, and detailed ranges of these conformational can be found in the supporting information as Table S3. The figures show that the distributions of monomeric TNFR1 are highly distinguishable from the distributions of dimeric TNFR1. More specifically, the simulation of TNFR1 *cis*-dimer forms narrower distributions along the conformational angles φ and b θ than the simulation of monomeric TNFR1, as shown by the red and black curves in Fig. S2a, b, respectively. This can be explained by the fact that *cis*-dimerization provides additional constraints to the conformational fluctuations of the receptor. As a result, TNFR1 in a *cis*-dimer might be less flexible and thus more prone to interact with the ligand.

After calculating the ranges of above conformational distributions and incorporating them into the equations in the Methods, we derived the binding rates for both ligand-receptor *trans*-interactions and *cis*-interactions between receptors under different states. The binding constants of *trans*-interaction between soluble TNFα and TNFR1 (sTNFα-TNFR1) were adopted from the data in previous literatures, in which the association rate equals 1.1 × 10^9^M^−1^ min^−1^ and the affinity equals 1.9 × 10^−11^M^−1^ ^48^. We converted these experimental measurements into the units used in our domain-based diffusion-reaction simulations based on a curve-fitting procedure implemented in our previous studies^49,50^. This gives an association rate of 0.04 ns^−1^ with a distance cutoff of 18 Å and a dissociation rate of 3.48 × 10^−13^ ns^−1^. Based on the calculated ranges of conformational parameters in Eq. (1), we further derived the association rate for the *trans*-interaction between membrane-bound ligand and receptor (mTNFα-TNFR1), which equals 0.1 ns^−1^. This result suggests that membrane confinement of the ligand facilitates its association to receptor due to the loss of conformational entropy. The association rate of trans-interaction between membrane-bound ligand and receptor is thus accelerated. Consequently, the binding affinity of the interaction further becomes 2.5 times stronger than the soluble ligand.

Unfortunately, detailed binding constants of *cis*-interactions between TNFR1 receptors have not been quantitatively characterized. As a result, a typical range of diffusion-limited rate constants from 10^5^M^−1^s^−1^ to 10^7^M^−1^s^−1^ was selected to represent the associate rate of *cis*-interaction in solution^51^. We converted these values into the units used in our domain-based diffusion-reaction simulations based on the curve-fitting procedure and further derived the 2D association rates of *cis*-interaction between two membrane-bound receptor monomers based on the calculated ranges of conformational parameters in Eq. (2). This gives the range of association rate from 4.7 × 10^−4^ ns^−1^ to 4.7 × 10^−6^ ns^−1^. Moreover, based on the calculated ranges of conformational parameters in Eqs. (3) and (4), we derived the association rates of *cis*-interaction between two sTNFα-TNFR1 complexes and between two mTNFα-TNFR1 complexes, respectively. Interestingly, our calculations show that the association rates of *cis*-interaction between two sTNFα-TNFR1 complexes range from 9.6 × 10^−3^ ns^−1^ to 9.6 × 10^−5^ ns^−1^, while the association rates of *cis*-interaction between two mTNFα-TNFR1 complexes range from 1.0 × 10^−1^ ns^−1^ to 1.0 × 10^−3^ ns^−1^.

Above results indicate that ligand binding might facilitate the *cis*-interaction between receptors, while the binding of membrane-bound ligands can make this *cis*-interaction even 10 times faster. The results can also be explained by an alternative possibility that the *cis*-interactions between monomeric TNFR1 facilitate ligand binding, while the membrane confinement of ligands can make this process even faster. Both mechanisms are due to the extra entropy loss after both ligand and receptor are constrained at the cell interface. In addition to association rates, the range of dissociation rate for the *cis-*interaction was taken between 10^−9^ ns^−1^ and 10^−13^ ns^−1^. Taken together, our tests cover the wide spectrum of dissociation constants from millimolar (mM) to nanomolar (nM), which binding affinities are within the typical range of ligand-receptor interactions in cell signaling systems^52^. All detailed values of our calculated binding rates and affinities can be found in Table S4 for both sTNFα-TNFR1 and mTNFα-TNFR1 systems. Given these binding parameters, the clustering of TNFR1 induced by soluble or membrane-bound TNFα was simulated by domain-based diffusion-reaction algorithm. Practically, association between a TNFα and a TNFR1 is triggered if the distance between their *trans*-binding sites is below the cutoff value. The probability to form a *trans*-interaction is further determined by calculated association rate. Relatively, the probability to break a ligand receptor interaction in a TNFα-TNFR1 complex is regulated by the *trans-*dissociation rate. In parallel, if the distance between the *cis*-binding sites of two TNFR1 is below the cutoff, association between these two receptors will be triggered, which probability is determined by the *cis*-association rate. Relatively, the probability to break two TNFR1 is regulated by the *cis* dissociation rate introduced above.

Other parameters in the simulations are specified as follows. The total length of a single simulation trajectory is 0.2 s (2 × 10^8^ns) with a time step of 10 ns. In the mTNFα-TNFR1 system, the length of each side along the two square surfaces is 577.3 nm. In the sTNFα-TNFR1 system, the same size was set for the surface at the bottom of the simulation box, while the height of the box is 100 nm. Additionally, the surface density of ligands and receptors is on the order of ~10^2^mol/µm^2^, which is within the typical range of experimental observation in T cells^53^. In specific, there are 50 trimeric TNFα ligands and 150 TNFR1 receptors in both sTNFα-TNFR1 and mTNFα-TNFR1 simulation scenarios. Based on the precise boundary element method^54^, the translational and rotational diffusion coefficients of TNFα in solvent were set to 72.6 μm^2^/s and 0.34°ns^−1^, respectively. On the other hand, the two-dimensional diffusions of TNFR1 and TNFα on plasma membrane are considered to be much slower due to the constraints of lipid bilayers. As a result, translational diffusion coefficient of 10μm^2^/s and rotational diffusion coefficient of 1°ns^−1^ were used based on our previous MD studies^55^. Moreover, we assume that diffusions of a TNFα-TNFR1 complex on membrane surface or interface are even slower, with translational and rotational coefficients of 5 μm^2^/s and 0.28°ns^−1^, respectively. When the size of a cluster continues to grow, its diffusions will become further slower and finally stop if it contains more than two full-size signaling complexes. Finally, 20 simulation trajectories were carried out for each system in both sTNFα-TNFR1 and mTNFα-TNFR1 scenarios to attain statistically meaningful results. All these trajectories were started from different randomly generated initial configurations.

**Fig. 4:**
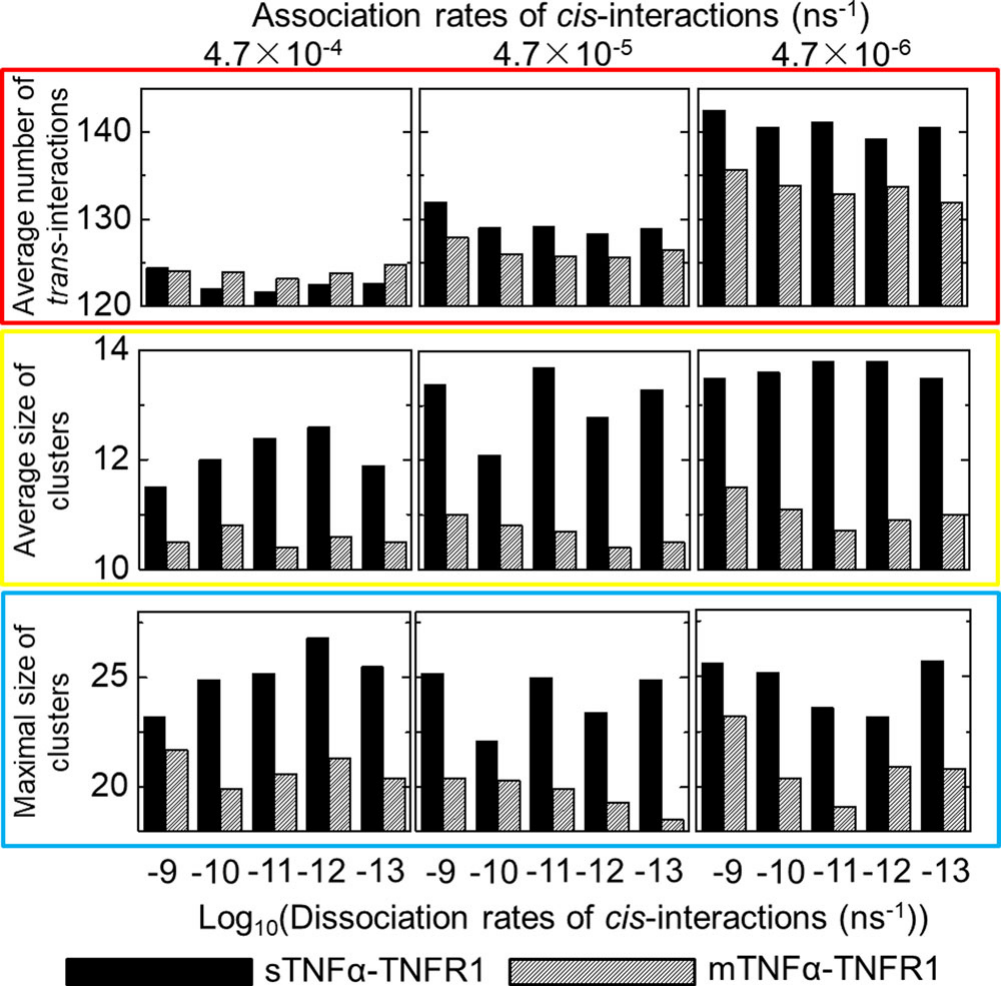
The results from domain-based diffusion-reaction simulations under different values of cis-interactions. Because binding constants of *cis*-interactions between TNFR1 receptors have not been experimentally characterized, different combinations of association and dissociation rates were tested in the domain-based simulations. For each combination, 20 simulation trajectories were carried out. We calculated the average number of ligand-receptor *trans*-interactions (red frame), as well as the average (yellow frame) and maximal (blue frame) size of clusters obtained from each combination after all simulations were terminated. The left, middle and right columns indicate the specific values of association rate, while dissociation rates used in the simulations are indexed at the bottom of each plot. The data derived from sTNFα-TNFR1 and mTNFα-TNFR1 systems are represented by black and striped bars, respectively.

After the termination of all these simulations, the number of ligand-receptor *trans*-interactions, the number of clusters and the size of each cluster were counted for each trajectory. Our calculated results are summarized as histograms in Fig. 4 under different association and dissociation rates of *cis*-interactions. The left, middle and right columns of the figure indicate different association rates used in each simulation, as denoted at the top of each column. Dissociation rates used in the simulations are indexed at the bottom of each plot. Data in the figure were collected from the last 2 × 10^7^ns of the corresponding simulation trajectories with an update of every 5 × 10^4^ns. Given the fact that 20 independent trajectories were carried out for each condition, all bars in the histograms of Fig. 4 corresponds to the statistical average of the last 8000 data points along the simulations. The average numbers of *trans*-interactions between ligands and receptors are shown by the upper row of Fig. 4, as highlighted by the red frame. The black bars represent the interactions formed by soluble ligands, while the striped bars represent the interactions formed by membrane-bound ligands. Interestingly, although the binding rates of *trans*-interactions under all conditions were fixed at the experimental value, our simulations show that the numbers of *trans*-interactions actually formed in various systems can be affected by the corresponding *cis*-interactions. Specifically, we found that the lower association rate of *cis*-interactions results in the higher number of *trans*-interactions. In other words, fast association between receptors prevents them from forming *trans*-interactions with their ligands.

Moreover, the average and maximal size of clusters formed along simulations are shown in by the middle and lower rows of Fig. 4, as highlighted by the yellow and blue frames respectively. The size of a cluster formed by ligand-receptor complexes is defined by the number of proteins which can be connected together through either *trans* or *cis*-interactions. A trimeric TNFα is counted as one protein, while a TNFR1 is also counted as one individual protein in a cluster. For instance, the schematic of a cluster shown in Figure S3 contains 4 ligand trimers (red) and 12 receptors (green). As a result, the size of the cluster is 16. Furthermore, the average size of clusters from a simulation trajectory is calculated as the average value of all clusters in the system. Relatively, the maximal size of clusters is the size of the largest cluster found in a given system. In all different combinations of *cis*-binding rates, Fig. 4 suggests that the clusters formed in sTNFα-TNFR1 system (black bars) are systematically larger than the clusters formed in mTNFα-TNFR1 system (stripe bars). The analysis from MD simulations suggests that after receptors form complexes with membrane-bound ligands, their lateral association can be strengthened. Surprisingly, our simulation results indicate that this enhanced *cis*-interaction plays a negative role in regulating ligand-receptor clustering. Detailed mechanism underlying this observation will be speculated below.

**Fig. 5:**
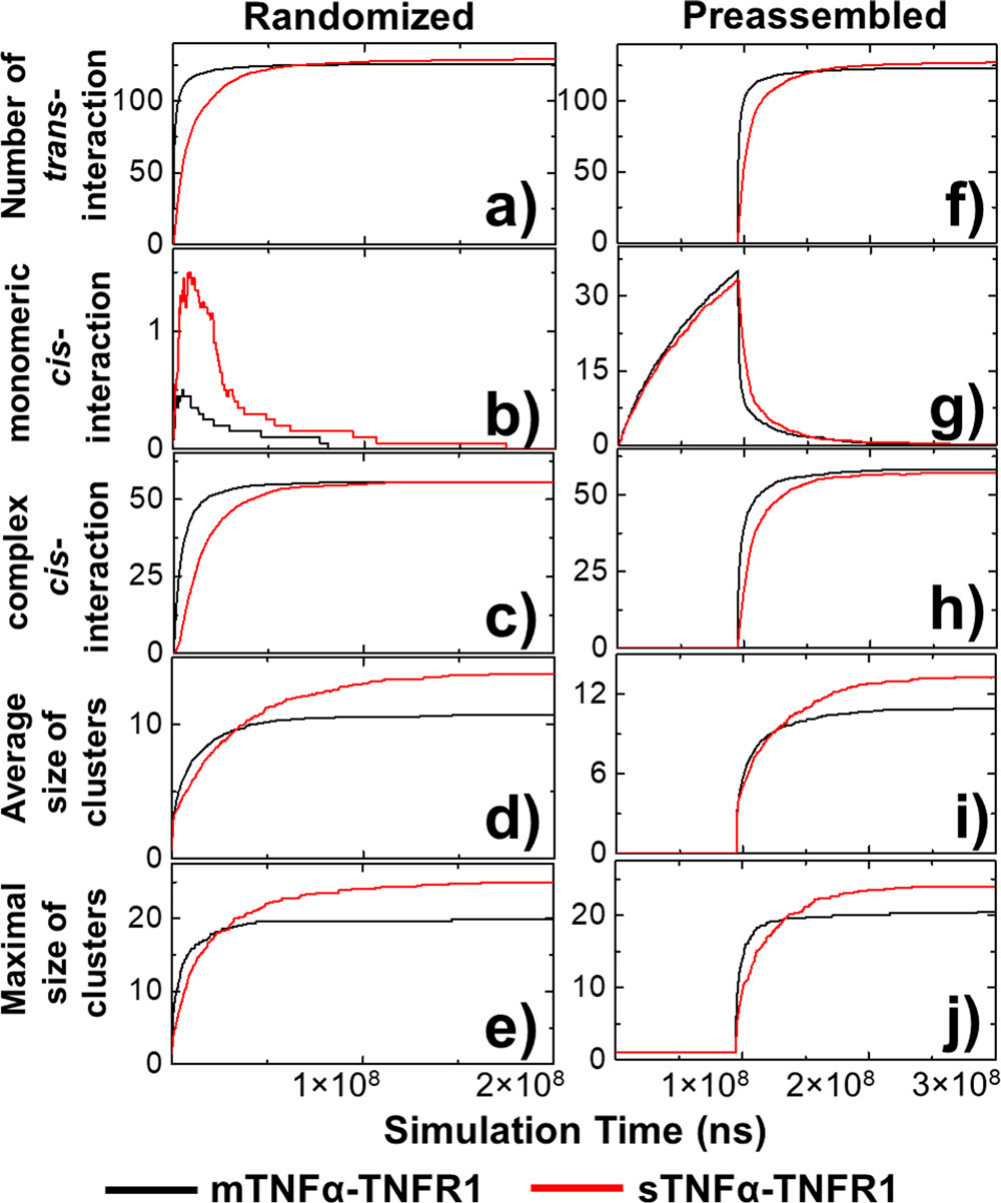
The comparison of kinetic profiles between simulations of soluble ligand and membrane-bound ligand. We compare the kinetic profiles averaged from sTNFα-TNFR1 system (red) with mTNFα-TNFR1 (black) system as a function of simulation time. These profiles include the average numbers of *trans*-interactions (**a**) among different trajectories; the average numbers of monomeric (**b**) and ligand-bound *cis*-interactions (**c**) among different trajectories; and the average (**d**) and maximal (**e**) size of clusters. Moreover, we tested an alternative starting model in which the ligand-receptor interactions were turned off at the beginning so that monomeric TNFR1 receptors can preassemble. The kinetic profiles from this alternative starting model were also plotted as a function of simulation time, including the average numbers of *trans*-interactions (**f**) among different trajectories; the average numbers of monomeric (**g**) and ligand-bound *cis*-interactions (**h**) among different trajectories; and the average (**i**) and maximal (**j**) size of clusters.

The kinetic profiles of receptor clustering were further compared between the sTNFα-TNFR1 and mTNFα-TNFR1 scenarios. For both scenarios, we selected the systems in which the association rates were fixed at 4.7 × 10^−5^ ns^−1^ and the dissociation rates were fixed at 1.0 × 10^−11^ ns^−1^. The obtained kinetic patterns are illustrated in Fig. 5 as a function of simulation time. The profiles of membrane-bound ligands are shown by the black curves, while the profiles of soluble ligands are shown by the red curves. All curves in the figure were averaged over all 20 trajectories. We first plotted the numbers of *trans*-interactions in Fig. 5a. The figure indicates that the system of mTNFα-TNFR1 simulation scenario reached equilibrium faster than the system of sTNFα-TNFR1 simulation scenario, which is based on the fact that the membrane confinement of ligands enhances its association with receptors. However, at the end of the simulations, slightly more *trans*-interactions were formed in the sTNFα-TNFR1 system than the mTNFα-TNFR1 system, as reflected in the histograms of Fig. 4. Additionally, the numbers of *cis*-interactions formed between monomeric receptors and formed between ligand-bound receptors were plotted in Fig. 5b, c, respectively. Figure 5b shows that in both systems, the number of *cis*-interaction between monomeric receptors reached a maximal level at the beginning and dropped to 0 by the end of the simulations. In the meantime, the numbers of *cis*-interaction between ligand-bound receptors increased and reached equilibrium, as shown in Fig. 5c. This suggests that the *cis*-interactions between ligand-bound receptors competed with the *cis*-interactions between monomeric receptors and then became dominant in the simulations after more and more receptors engaged with ligands. Different from the mTNFα-TNFR1 system, the monomeric *cis*-interaction in the sTNFα-TNFR1 system reached a much higher level and then decayed more slowly, while the *cis*-interactions between ligand-bound receptors in the sTNFα-TNFR1 system also reached equilibrium more slowly. Finally, the average and maximal size of clusters formed along simulations are shown in Fig. 5d, e, respectively. The figures show that while the kinetics of clustering in the sTNFα-TNFR1 system is slower, larger clusters were obtained at the end of the simulations than the mTNFα-TNFR1 system. It is worth mentioning that the size of clusters observed in our simulations is directly comparable to a recent single-molecule image experiment^12^.

As we mentioned earlier, TNFR1 can preassemble on cell surface via PLAD regions prior to ligand binding. This ligand-independent dimerization of receptors has been recently observed by fluorescence resonance energy transfer (FRET)^56,57^. In order to address this issue, we adopted an alternative simulation strategy in which TNFα were not included at the beginning, but only introduced into the system after the simulations reached 9.5 × 10^7^ ns. Therefore, during the first 9.5 × 10^7^ ns monomeric receptors had the opportunity to preassemble before they expose to ligands. The total length of simulation trajectories using this new strategy is 0.3 sec (3 × 10^8^ns), consisting the first 9.5 × 10^7^ ns for receptor preassembly and the next 2.05 × 10^8^ ns for ligand-induced receptor clustering. The detailed kinetic patterns generated from this alternative model are summarized from Fig. 5f–j. Especially, Fig. 5g shows the rapid increase of *cis*-interactions between monomeric receptors before they exposed to ligands. This dimerization of TNFR1 monomers on cell surface, however, decayed soon after the introduction of ligands, whereas the numbers of ligand-induced *cis-*interactions started to increase, as shown in Fig. 5h. This result demonstrates that the preassembled receptors have been suppressed by the *cis-*interactions between ligand-bound receptors. In terms of the differences between the sTNFα-TNFR1 and mTNFα-TNFR1 systems, similar phenomena were observed: smaller clusters were more likely to form by receptors if they engaged with membrane-bound ligands (Fig. 5i, j). Our tests thereby confirmed that in receptor preassembled systems, sTNFα-TNFR1 complexes still has the higher probability to form large clusters than mTNFα-TNFR1 complexes.

In the sTNFα-TNFR1 simulation system, decreasing the height of the simulation box will increase the concentration of soluble ligand in the extracellular space, leading to the result that ligands and receptors are more likely to interact with each other and form larger cluster size. On the contrary, increasing the height of the simulation box will decrease the concentration of soluble ligand in the extracellular space, resulting in smaller cluster size. In our current sTNFα-TNFR1 system, the height of the box is 100 nm, while in the current mTNFα-TNFR1 system, the distance between the two lipid bilayers equals 8 nm. Therefore, if we apply simulation of sTNFα-TNFR1 to a system which height is identical to the distance between two lipid bilayers in the mTNFα-TNFR1 system, we need to lower the height in our current simulation box, which means the concentration of soluble ligand will further increases. As a result, the soluble ligands will have a higher probability to interact with receptors, resulting in even larger size of sTNFα-TNFR1 clusters.

**Fig. 6:**
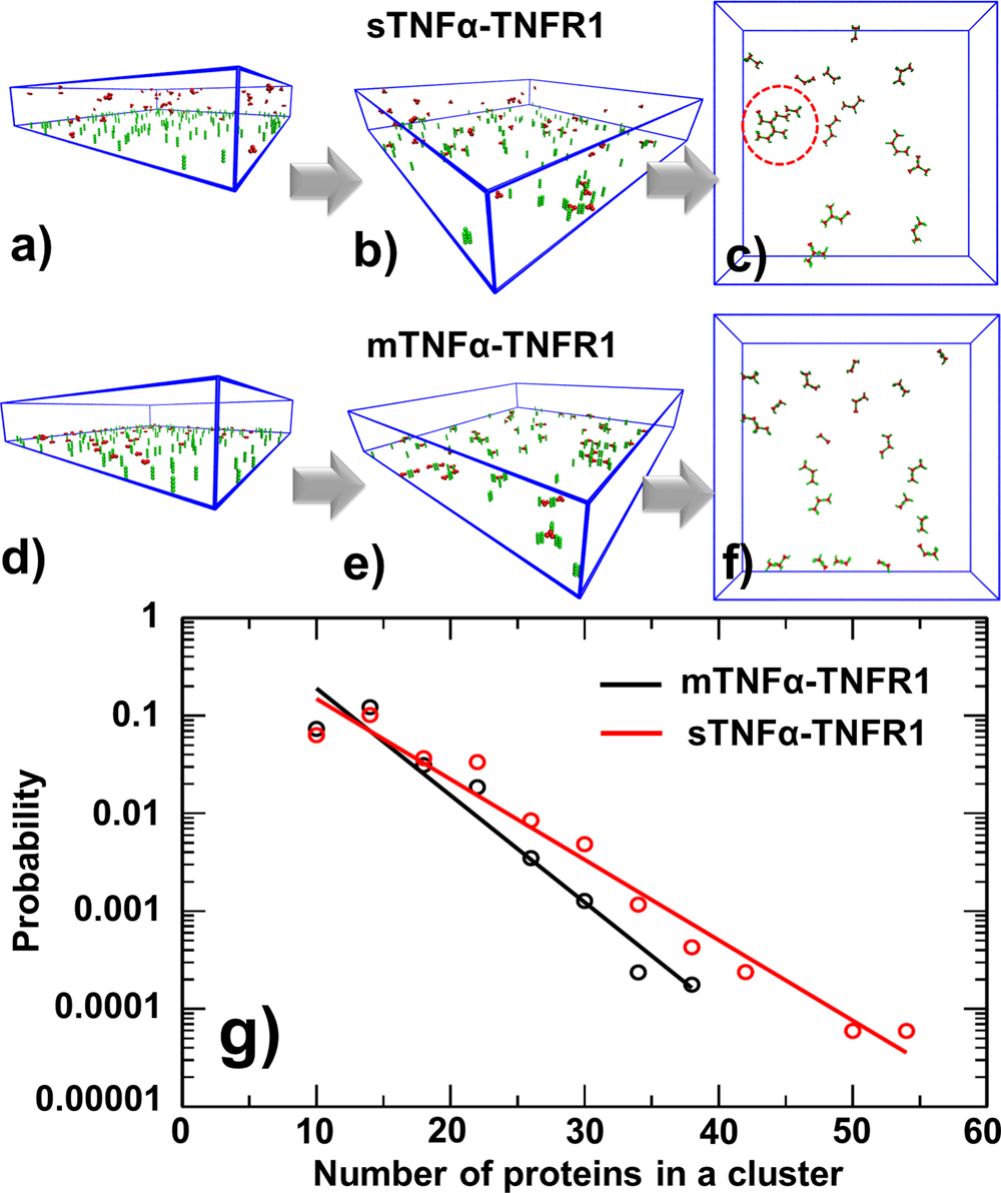
The representative snapshots and cluster size distributions generated by domain-based diffusion-reaction simulations. Some representative snapshots were selected from the simulations to visualize the spatial process of clustering. Specifically, the initial (**a**), middle (**b**) and final (**c**) configurations along a trajectory in sTNFα-TNFR1 system are compared with the initial (**d**), middle (**e**) and final (**f**) configurations along a trajectory in mTNFα-TNFR1 system. We found that large clusters can be organized into hexagonal lattice, as highlighted by the red dashed circle. Finally, we compared the cluster size distributions (**g**) in sTNFα-TNFR1 system (red) to the distribution in mTNFα-TNFR1 system (black). Given the logarithmic scale of the y-axis, the distributions of cluster size in both systems can be fitted by a single exponential function, whereas the clusters formed by sTNFα-TNFR1 complexes have the feasibility to grow into larger sizes.

Some representative snapshots were selected from the simulations to visualize the spatial process of clustering. Specifically, the initial, middle and final configurations along a trajectory of sTNFα-TNFR1 simulation scenario were plotted in Fig. 6a, b and c, respectively. Relatively, the initial, middle and final configurations along a trajectory of mTNFα-TNFR1 simulation scenario were plotted in Fig. 6d–f, respectively. The comparison of the final configurations confirmed that clusters formed by sTNFα-TNFR1 complexes can be remarkably larger than the clusters formed by mTNFα-TNFR1 complexes, as highlighted by the red dashed circle in Fig. 6c. Moreover, we found that these clusters are organized into hexagonal lattice through the combination of three-fold symmetry in trimeric TNFα ligand and the two-fold symmetry of the *cis*-interaction between TNFR1 receptors. This spatial pattern was also previously suggested by others based on their experimental evidences^13^. The specific configurations of some large clusters formed by sTNFα-TNFR1 complexes in different trajectories were selected and plotted in Figure S4. The size of the first selected cluster equals 36, consisting of 9 ligand trimers and 27 receptors, as shown in Figure S4a. The size of the second selected cluster equals 30, consisting of 8 ligand trimers and 22 receptors, as shown in Figure S4b. The size of the third selected cluster equals 33, consisting of 9 ligand trimers and 24 receptors, as shown in Figure S4c. The figure indicates that hexagonal lattice like structures were formed in all three clusters.

We compared the cluster size distribution in sTNFα-TNFR1 system to the distribution in mTNFα-TNFR1 system, as shown in Fig. 6g. The distributions were derived by counting the number of proteins in each cluster at the end of all trajectories and summed from all combinations of *cis*-binding rates. The probability distribution of sTNFα-TNFR1 system is shown by red dots as a function of cluster size, while the probability distribution of mTNFα-TNFR1 system is shown by black dots. Given the logarithmic scale of the y-axis, the figure indicates that the distributions of cluster size in both systems can be fitted by a single exponential function. Moreover, the size distribution functions confirm that mTNFα-TNFR1 system tend to have higher probability to form clusters with smaller sizes. On the other hand, clusters formed by sTNFα-TNFR1 complexes have the feasibility to grow into much larger sizes. A student’s *t*-test was also performed to the two distributions. Both systems contain more than 4 × 10^3^ clusters. The average size of clusters formed in sTNFα-TNFR1 system equals 13.6 and the standard deviation of the distribution is 5.1. The average size of clusters formed in mTNFα-TNFR1 system equals 12.1 and the standard deviation of the distribution is 3.8. The calculated t-score equals 14.5 and the corresponding P-value is lower than 0.00001. As a result, the null hypothesis that no difference exists between two sets can be rejected with a 95% confidence interval, which suggests that the size of clusters induced by soluble ligands is significantly larger than the clusters induced by membrane-bound ligands and this difference is statistically meaningful. Unfortunately, the difference between sTNFα-induced and mTNFα-induced clustering have not been previously reported. It would be interesting if this computational observation can be validated by experimental approaches such super-resolution imaging.

The mechanism underlying our observation that larger clusters are preferred in sTNFα-TNFR1 system than mTNFα-TNFR1 system is speculated as follows. In detail, the differences in clustering are regulated by the binding rates of *trans*- and *cis*-interactions. These rates are mediated by the conformational fluctuations of ligand and receptor, while membrane confinements can bring additional constraints to the systems. The higher ratio of conformational fluctuations embedded in the mTNFα-TNFR1 system, as reflected in Eq. (4), leads into the result that the *cis*-interaction between receptors becomes much more strengthened after they engage with membrane-bound ligands, comparing with the *cis*-interaction between monomeric receptors before ligand binding. As a result, complexes between membrane-bound TNFα and TNFR1 are more easily to aggregate and then kinetically trapped in these rapidly formed small clusters. Unlike mTNFα-TNFR1 system, the clusters formed by TNFR1 and soluble TNFα are more variable. Reflected by the relatively lower ratio of conformational fluctuations embedded in Eq. (3), the *cis*-interaction between ligand-bound TNFR1 receptors is less strengthened. Moreover, the association between sTNFα and TNFR1 is relatively slower due to the higher degrees of freedom in the soluble ligand. This leads to the result that a slower but more dynamic equilibrium can remain throughout the simulation in which clusters have higher probabilities to be reorganized. Consequently, this higher level of dynamics ensures that small clusters in sTNFα-TNFR1 system are more likely to dissolve, merge and grow into clusters with larger size.

In many various cellular signaling systems, it has been revealed that the formation of high-order ligand-receptor aggregates can promote a threshold-like signal response in which the signaling pathways can only be turned on under a persistent and high dose of external stimulation^58^. In the case of TNFα-mediated signaling pathways, the assembly of TNFα-TNFR1 clusters further provides spatial platform to downstream signaling molecules such as TNF receptor 1 associated protein with death domain (TRADD)^59^ or TNF receptor associated factors (TRAF)^60^ through the interactions with receptor’s cytoplasmic region. While TRAF’s C-terminal domain maintains interactions with TNFR1, its N-terminal regions function as the platform of poly-ubiquitination^61^. The ubiquitination leads to the degradation of IκB which is an inhibitory factor of nuclear factor kappa-light-chain-enhancer of activated B cells (NF-κB)^62,63^. NF-κB is an important transcription factor regulates the stimulation of inflammatory responses^64–67^. Degradation of IκB by ubiquitination can released NF-κB from cytoplasm to cell nucleus and further turn on its target genes. Using a hybrid computational model which combines diffusion-reaction algorithm with stochastic simulation of chemical reactions, we recently showed that the oscillatory responses in NF-κB signaling network were maintained only when receptors were assembled into clusters with reasonably large sizes^68^. Considering our simulation results suggest binding of soluble TNFα is more likely to trigger the formation of large ligand-receptor clusters than membrane-bound TNFα, current study therefore provides insights about why the activation of TNFR1-mediated signaling pathways is preferentially through the soluble form of TNFα instead of the membrane-bound form.

In living systems, binding of TNFα to TNFR1 can not only activate proinflammatory NF-κB pathway, but also can lead to cell death by triggering apoptosis and necroptosis signaling^69^. However, cell death signaling is typically inhibited by protein complexes such as a TRAF2 trimer and a single cellular inhibitor of apoptosis-1 (cIAP1) or cIAP2 E3 ligase molecule^70^. Moreover, TNFα binds to another member in TNFR superfamily, TNFR2, in addition to TNFR1. Different from TNFR1, TNFR2 only responds to membrane-bound TNFα, but not its soluble form^71^. Interestingly, it has been found that the signaling outcome of different TNFR1-mediated pathways can be modulated by TNFR2. For instance, activation of TNFR2 can lead to a significant depletion of cytosolic TRAF2-cIAP1/2 complexes, thus switching the TNFR1-mediated signaling to cell death^72^. We speculate that the differences in TNFR1 and TNFR2’s responds to membrane-bound and soluble ligands are resulted from the conformational dynamics of the two receptors, which further affects the kinetics of their ligand binding and clustering. This can be tested by our multiscale simulations in the future. It is also possible to extend our current hybrid model to study the crosstalk between TNFR1-mediated proinflammatory and apoptosis signaling pathways, differentiate the signaling outcomes induced by membrane-bound or soluble TNFα, and understand the interplay between TNFR1 and TNFR2-mediated signaling. Finally, our methods can be applied to study clustering of ligand-receptor complexes for other members in TNFR superfamily, which can be classified into two categories^16^. Members in the first category, including TNFR1, are robustly activated by soluble ligands, while members in the second category, such as 4-1BB, CD27, CD40 and CD95, only response to membrane-bound ligands. This might be resulted from the difference between soluble ligand-induced and membrane-bound ligand-induced clustering for each specific receptor in the superfamily. A systematic test using our computational simulation in the future can help understanding the underlying mechanisms.

In summary, we compared receptor clustering induced by soluble ligands to receptor clustering induced by the same ligands, but confined on the membrane surface. Specifically, using the interactions between ligand TNFα and receptor TNFR1 as an example, we first analyzed the conformational dynamics of two systems: sTNFα-TNFR1 and mTNFα-TNFR1, by all atom MD simulations. We found that membrane confinement of ligands introduces extra constraints not only to their own fluctuations, but also to the entire ligand-receptor complexes. This loss of conformational entropy in turn enhances the *trans*-interaction between receptors and membrane-bound ligands, as well as the *cis-*interactions between two mTNFα-TNFR1 complexes. Consequently, the results from our domain-based diffusion-reaction simulations indicate that much smaller clusters will be formed if TNFα ligands are confined on cell surface. In contrast, the clustering triggered by soluble TNFα is more dynamic, and the size of clusters formed after simulations is consistently larger. This provides explanation to the experimental observation in which TNFR1 was preferentially activated by soluble TNFα. Our study, therefore, demonstrated the impact of ligand confinement on dynamics of receptor clustering.

## Methods

### A domain-based diffusion-reaction simulation for TNF ligand-receptor clustering

Recent experimental discoveries suggested that TNFα can induce the clustering of TNFR1 on plasma membrane of living cells^12^. We applied a diffusion-reaction algorithm to simulate this process^73^. The algorithm is based on a domain-based coarse-grained model^74^. In this coarse-grained model, the molecular geometry of TNF ligand and receptor was specifically designed to describe their corresponding structural arrangement. For an example, the THD domain of each subunit in a trimeric TNF ligand^75^ is represented by a spherical rigid body which radius equals 3 nm. Subsequently, three rigid bodies in a ligand trimer are spatially constricted together with a three-fold symmetry. On the other hand, there are four consecutive repeats of cysteine-rich domains (CRDs) in the extracellular regions of receptors TNFR1^4^. These four CRD domains are also coarse-grained into spherical rigid bodies and further straightly aligned into rod-like shape. The radius of each CRD rigid body equals 2 nm. Additionally, TNFα and TNFR1 are allowed to form a *trans*-interaction, while two TNFR1 are allowed to form a *cis*-interaction. As shown by the yellow dots in Fig. 1a, binding sites for the *trans*-interaction are assigned to each ligand subunit, as well as on the surface of the second domain in each receptor. Similarly, as shown by the blue dots in Fig. 1a, binding sites for the *cis*-interactions are assigned on the surface of the first domain in each receptor. Moreover, the cis-binding sites are on the opposite side of *trans*-binding sites, so that they can coexist to allow high-order aggregation of ligand-receptor complexes.

Given the model representation, we designed two scenarios to explicitly estimate how membrane confinement of TNFα ligands impacts TNFR1 receptor clustering. These scenarios specifically compare the receptor clustering induced by soluble ligands to the receptor clustering induced by membrane-bound ligands. The system of soluble ligands (sTNFα) was first constructed in the first scenario. As shown in Fig. 1b, the plasma membrane of a cell is represented by the bottom surface of a three-dimensional simulation box, while the space above the plasma membrane represents the extracellular region. As the initial configuration of simulation, TNFR1 receptors are randomly placed on the plasma membrane, while TNFα ligands are distributed in the extracellular region. In contrast, the system of membrane-bound ligands (mTNFα) was constructed in the second scenario. As shown in Fig. 1c, the interface between two cells and is modeled as two layers of flat surfaces overlapping on top of each other. Receptors are randomly placed on the lower bound of the interface, while their ligands form random distributions on the opposite side of surface layer.

Following the initial configuration, the dynamics of the system is evolved as follows. Within each simulation time step, ligands and receptors are first selected by random order for stochastic movements. Specifically, the probability and amplitude of translational and rotational movements are determined by the corresponding diffusion coefficient of each ligand and receptor. Diffusions of TNFR1 are confined within plasma membrane. On the other hand, the sTNFα in the first scenario are free to move throughout the simulation box, while the mTNFα in the second scenario are only allowed to diffuse within plasma membrane. Periodic boundary condition is applied to move the molecules along x and y directions of both lipid bilayer in the second scenario. As for the movements of ligands along z direction in the first scenario, they will be bounced back if moving beyond the top of the simulation box or below the plasma membrane. If a ligand binds to a receptor, the entire complex will move as a single unit either on plasma membrane as in the first scenario, or on the cell interface as in the second scenario. Similarly, if two receptors form a lateral dimer, they will also move together. After diffusions, new interactions could form among receptors or between ligands and receptors under their newly updated configuration, while preexisting bonds might be broken. We analyze the binding kinetics of all *trans*- and *cis*-interactions in the system, based on their association and dissociation rates as described in the next section. The iteration of above diffusion-reaction process will not be terminated until the end of simulations or the spatial patterns in corresponding system reach equilibrium.

### Linking trans- and cis- binding rates between membrane-bound and soluble systems

In above domain-based simulation, each TNFα ligand in the first scenario can freely diffuse along three translational and three rotational degrees of freedom in the extracellular region. In contrast, in the second scenario, diffusions of ligands are constricted in the plasma membrane, which is a two-dimensional system. This restriction of motions can cause unneglectable impacts on kinetic properties of binding between ligands and receptors. Unfortunately, the kinetics of binding between proteins in these 2D membrane environments is difficult to measure with current experimental techniques. While association rates are closely regulated by molecular diffusion and thus are concentration dependent and sensitive to different cellular environments, dissociation rates are fully relied on the strength of interactions, especially the short-range interactions, between residues cross the interfaces of a protein complex^76^. Therefore, it is reasonable to assume that membrane confinements only affect the process of association between ligand and receptor, but not their dissociation. Based on our previous theoretic analysis using statistical thermodynamics^77,78^, we can estimate the effects of membrane confinement on conformational fluctuations of ligand and further on the binding to its receptor. In detail, the relation between the association rate of a *trans*-interaction formed at cell interface and the rate of the same interaction formed in solution has the following form.

1 $$\frac{{k}_{on}^{trans}({L}_{M}/{R}_{M})}{{k}_{on}^{trans}({L}_{S}/{R}_{S})}=\frac{8{\pi }^{2}\times (\varDelta {\omega }_{{L}_{M}/{R}_{M}}\times \varDelta {h}_{{L}_{M}/{R}_{M}})}{(\varDelta {\omega }_{{L}_{M}}\times \varDelta {h}_{{L}_{M}})\times (\varDelta {\omega }_{{R}_{M}}\times \varDelta {h}_{{R}_{M}})}$$

In Eq. (1), *L*_*M*_, *R*_*M*_*, L*_*S*_ and *R*_*S*_ stand for the membrane-bound ligand, membrane-bound receptor, soluble ligand and soluble receptor, respectively. *L*_*M*_/*R*_*M*_ and *L*_*S*_/*R*_*S*_ indicate a ligand-receptor complex formed through the *trans*-interaction at 2D cell interface or in 3D solution, respectively. The parameter Δh represents the range of conformational fluctuations for a corresponding membrane-bound molecule along its membrane normal, while Δω corresponds to the volume in the rotational phase space of the molecule after membrane confinement. The association rate of protein interactions in solution can be easily measured by traditional experimental approaches such as surface plasmon resonance (SPR). As a result, by analyzing the values of conformational parameters in above equation, we will be able to calculate the association rate of ligand-receptor interaction at 2D cell interface.

In addition to the *trans*-interaction, the *cis*-interactions can be formed in real cellular environments between TNFR1 which are also anchored on plasma membrane. With the same mechanism, the association rate of a *cis*-interaction formed between TNFR1 on 2D cell surface can be linked to the rate of the same interaction formed in 3D solution by the following equation.

2 $$\frac{{k}_{on}^{cis}({R}_{M}-{R}_{M})}{{k}_{on}^{cis}({R}_{S}-{R}_{S})}=\frac{8{\pi }^{2}}{(\varDelta {\omega }_{{R}_{M}}\times \varDelta {h}_{{R}_{M}})}$$

In Eq. (2), *R*_*M*_-*R*_*M*_ and *R*_*S*_-*R*_*S*_ indicate a *cis*-dimer formed between two membrane-bound TNFR1 receptors or a *cis*-dimer formed between two soluble TNFR1 receptors, respectively. Moreover, we speculate that the conformational dynamics of receptors in their monomeric state is different from the ligand-bound states. Intuitively, there are less constraints in the dynamics of a monomeric receptor, comparing to a ligand-receptor complex in which all three receptors are tethered to plasma membrane. This difference in the configurational entropy between monomeric and ligand-bound receptors can further affect the association rate of their *cis*-interactions. As a result, we can theoretically derive the 2D association rates of a *cis*-interaction between two ligand-bound receptors from the 2D association rates of a *cis*-interaction between two monomeric receptors, which can be calculated from Eq. (2). Finally, the membrane-bound ligands can result in additional constrains to the complex relative to the soluble ligands. At cell interface, both ends of a complex formed by mTNFα and TNFR1 are anchored to lipid bilayers. Consequently, the association rate of a *cis*-interaction between ligand-bound receptors at cell interface should also be distinguishable from the association rate of a *cis*-interaction between ligand-bound receptors formed on a single layer of plasma membrane. Specifically, association rates for these two types of *cis*-interactions can be written by the following two equations.

3 $${k}_{on}^{cis}({L}_{S}/{R}_{M}-{L}_{S}/{R}_{M})={k}_{on}^{cis}({R}_{M}-{R}_{M})\times \frac{(\varDelta {\omega }_{{R}_{M}}\times \varDelta {h}_{{R}_{M}})}{(\varDelta {\omega }_{{L}_{S}/{R}_{M}}\times \varDelta {h}_{{L}_{S}/{R}_{M}})}$$

4 $${k}_{on}^{cis}({L}_{M}/{R}_{M}-{L}_{M}/{R}_{M})={k}_{on}^{cis}({R}_{M}-{R}_{M})\times \frac{(\varDelta {\omega }_{{R}_{M}}\times \varDelta {h}_{{R}_{M}})}{(\varDelta {\omega }_{{L}_{M}/{R}_{M}}\times \varDelta {h}_{{L}_{M}/{R}_{M}})}$$

The symbol *L*_*S*_/*R*_*M*_* − L*_*S*_/*R*_*M*_ in Eq. (3) indicates a *cis*-dimer formed between two complexes in which ligands are soluble and only receptors are on the plasma membrane (sTNFα-TNFR1), while the symbol *L*_*M*_/*R*_*M*_* − L*_*M*_/*R*_*M*_ in Eq. (4) indicates a *cis*-dimer formed between two complexes in which both ligands and receptors are attached on the plasma membrane in cell interface (mTNFα-TNFR1). Having these association rates, we can calculate the dissociation rates based on the binding affinity of the corresponding *trans-* or *cis-*interactions. All these rate constants will in turn be fed into the domain-based diffusion-reaction simulation. Therefore, all-atom MD simulations were utilized to sample the conformational space of ligand and receptor under different conditions, so that the values of all conformational parameters in above equations can be estimated in order to calculate the relevant association rates.

### Deriving conformational dynamics of membrane-bound systems by MD simulations

The atomic coordinates of trimeric THD domains from TNFα were obtained from the crystal structure with PDB id 3ALQ. Because no experimental structure is currently available for the complex between TNFα and TNFR1, the initial structure of the complex, except their corresponding transmembrane domains and linker regions, was adopted from the computational model that was previously built by Xie’s group^79^. For monomeric TNFR1, there are two experimental structures available. Comparing to the one with PDB id 1NCF which was used in our simulations of TNFR1 *cis*-dimer, the last CDR domain in the other with PDB id 1EXT is structurally more complete. Additionally, the model of TNFα-TNFR1 complex was previously constructed on the basis of TNFR1 (PDB id 1EXT) and TNFα-TNFR2 complex (PDB id 3ALQ). To be consistent with the simulations of TNFα-TNFR1 complex, we decided to adopt the atomic coordinates of TNFR1’s extracellular domains from 1EXT. It is worth mentioning that the receptor in 1EXT exists as an anti-parallel dimer, which is not compatible with the parallel assembly observed in 1NCF. This anti-parallel binding interface was not included in the following simulations. Moreover, the structure of TNFα-TNFR1 complex could also be modeled by using the crystal structure of TNFβ-TNFR1 complex (PDB id 1TNR) as a basis to guide the superposition of trimeric TNFα. However, because the structures of TNFα-TNFR2 and TNFβ-TNFR1 complexes share high similarity, we believe the model outcome should be independent to alignment method.

Subsequent to the extracellular regions, the transmembrane domains of TNFR1 and mTNFα in the relevant systems were built as standard α-helices, and the linker regions between the transmembrane and extracellular domains of the ligand and receptor were modeled by the online server, I-TASSER^80^ and ModLoop^81^. The transmembrane domain of each receptor was further embedded in a lipid bilayer comprised of around 500 POPC molecules. Similarly, the three transmembrane domains of a mTNFα ligand were also inserted in a lipid bilayer comprised of around 500 POPC molecules. Moreover, a model of double lipid bilayers was built to hold the mTNFα-TNFR1 complex. The average distance between the two lipid bilayers equals around 8 nm. Finally, counter-ions (Na^+^, Cl^−^) were added in all above systems to neutralize the net charge in the simulation box and to maintain an appropriate ionic strength (0.1 M).

Atomistic simulations were carried out using GROMACS with the CHARMM36m force field for proteins, CHARMM36 force field for lipids and TIP3P water. Initially, the systems were energy minimized with the algorithm of steepest descent until the maximum force was lower than 1000 kJ mol^−1^ nm^−1^ on each atom. Then the systems were equilibrated under NVT and NPT conditions for 6 ns respectively, with a position restraint applied to all heavy atoms of the proteins, so that the solvent (water/lipids) was able to re-orient themselves around the protein. Covalent bonds were constrained using the LINCS algorithm, and an integration time step of 2 fs was used together with the leapfrog integrator. A cutoff of 12 Å was used for van der Waals interactions, and electrostatic interactions were calculated with the particle mesh technique for Ewald summations, also with a cutoff of 12 Å. Temperature and pressure are controlled using the v-rescale thermostat (τT = 0.1 ps) and the Parrinello-Rahman barostat (τP = 0.1 ps), respectively. A 400 ns trajectory was generated for the TNR1 monomer, while a 300 ns trajectory was generated for the mTNFα trimer. For the mTNFα-TNFR1 system in which the complex was restrained by two membranes bilayers, the production run was performed for 300 ns. For the sTNFα-TNFR1 system with a single membrane bilayer, a 500 ns trajectory was generated. Finally, for the system with a *cis*-dimer of TNFR1, a 300 ns trajectory was generated. In summary, the total all-atom MD simulation time for this study is 1.8 microseconds. An overview of the simulation systems in this study can be found in Table S1.

### Reporting summary

Further information on research design is available in the Nature Research Reporting Summary linked to this article.

## Supplementary information

Supporting Information

Reporting Summary

## Data Availability

All the source data and relevant information can be obtained by contacting the corresponding author upon reasonable request.

## Code availability

All the source codes for domain-based diffusion-reaction simulation can be found in the GitHub repository: https://github.com/xiaopuren/membraneTNFaR1.
