# Supplementary material for: Understanding the functional role of membrane confinements in TNF-mediated signaling by multiscale simulations
Source: Commun Biol. 2022 Mar 11;5:228. doi: 10.1038/s42003-022-03179-1 (PMC8917213; doi:10.1038/s42003-022-03179-1)
Supplement: Supplementary file 1 — Supporting Information [file 42003_2022_3179_MOESM1_ESM.pdf]

Understanding the Functional Role of Membrane Confinements in TNF-mediated Signaling by Multiscale Simulations

Supporting Information

Zhaoqian Su[1], Kalyani Dhusia[1], and Yinghao Wu[1,*]

[1]Department of Systems and Computational Biology, Albert Einstein College of Medicine, 1300 Morris Park Avenue, Bronx, NY, 10461, USA

Table S1: An overview of the simulated systems.

System	Force Field	Temperature	Time	Number of water molecules	Number of POPC lipids
**TNFR1**	CHARMM36m	310K	400 ns	79k	442
**sTNFα-TNFR1 complex**	CHARMM36m	310K	500 ns	78k	554
**mTNFα**	CHARMM36m	310K	300 ns	75k	554
**mTNF-TNFR1 Complex**	CHARMM36m	310K	300 ns	188k	1108
**TNFR1-dimer**	CHARMM36m	310K	300ns	86k	500

Table S2: Protein fluctuation and binding parameters for TNFR1 receptor, sTNFα-TNFR1 complex, mTNFα and mTNFα -TNFR1 complex

	TNFR1 receptor	sTNFα-TNFR1 complex	mTNFα	mTNFα -TNFR1 complex
Δh	24 Å	13 Å	11 Å	5.8Å
Δφ	180°	55°	31°	177°
Δθ	40°	33°	18°	4.8°
Δψ	128°	54°	32°	180°

Table S3: Protein fluctuation and binding parameters for monomeric and dimeric TNFR1 receptor

	TNFR1	TNFR1-dimer
Δh	12 Å	12 Å
$\Delta \varphi$	94.3°	65.2°
$\Delta \theta$	20.3°	14.7°
$\Delta \psi$	64.2°	64.7°

Table S4: Binding parameters in KMC simulation

	sTNFα-TNFR1	mTNFα-TNFR1
R-L Asso Rate	0.04	0.1
R-L Diss Rate	3.48e-13	3.48e-13
Monomer Cis Asso Rate	4.7e-4 ~ 4.7e-6	4.7e-4 ~ 4.7e-6
Monomer Cis Diss Rate	1.12e-09 ~ 1.12e-13	1.12e-09 ~ 1.12e-13
Complex Cis Asso Rate	9.6e-3 ~ 9.6e-5	e-1 ~ e-3
Complex Cis Diss Rate	1.12e-09 ~ 1.12e-13	1.12e-09 ~ 1.12e-13

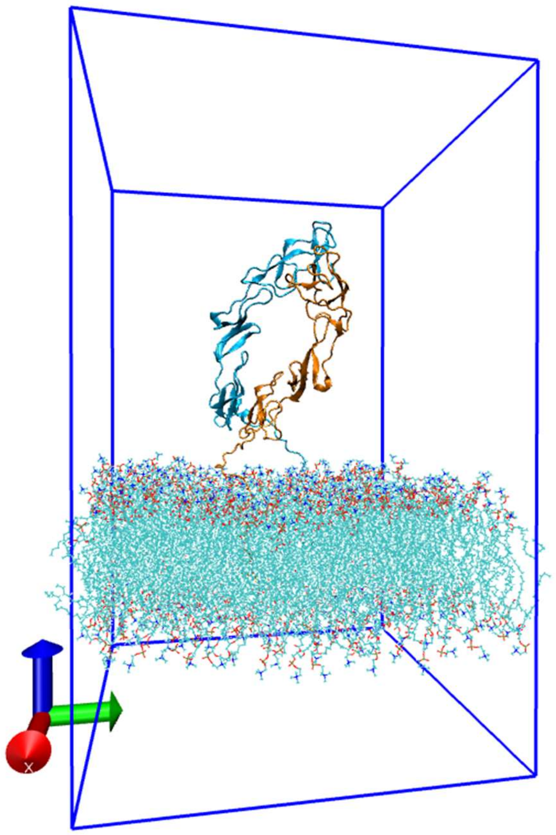

Figure S1: the simulation setup for a *cis*-dimer of TNFR1 on lipid bilayer

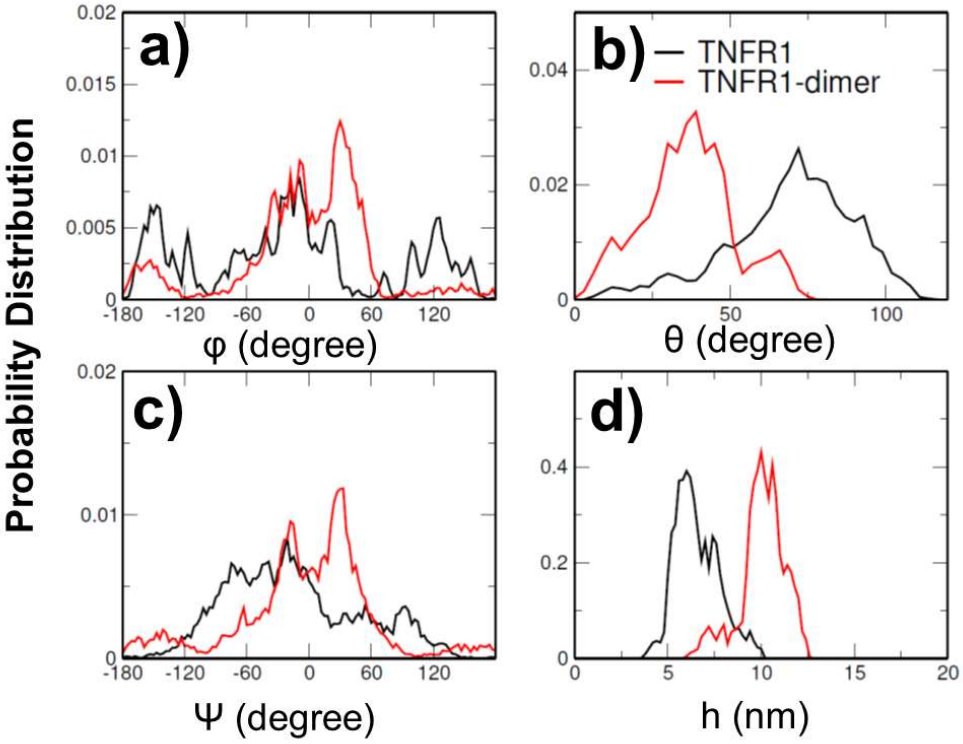

Figure S2: the comparison of distributions for all conformational parameters between TNFR1 monomer and TNFR1 *cis*-dimer. As indexed by curves with black for TNFR1 monomer and red for TNFR1 dimer, the distributions of the angle around the long principal axis z′ of the protein ψ are shown in **(a)**; the distributions of the tilting angle between this principal axis and the membrane normal θ are shown in **(b)**; and the distribution of the angle around the membrane normal z φ are shown in **(c)**. Similarly, detailed distributions of translational fluctuations are shown in **(d)** for proteins in four modeled systems.

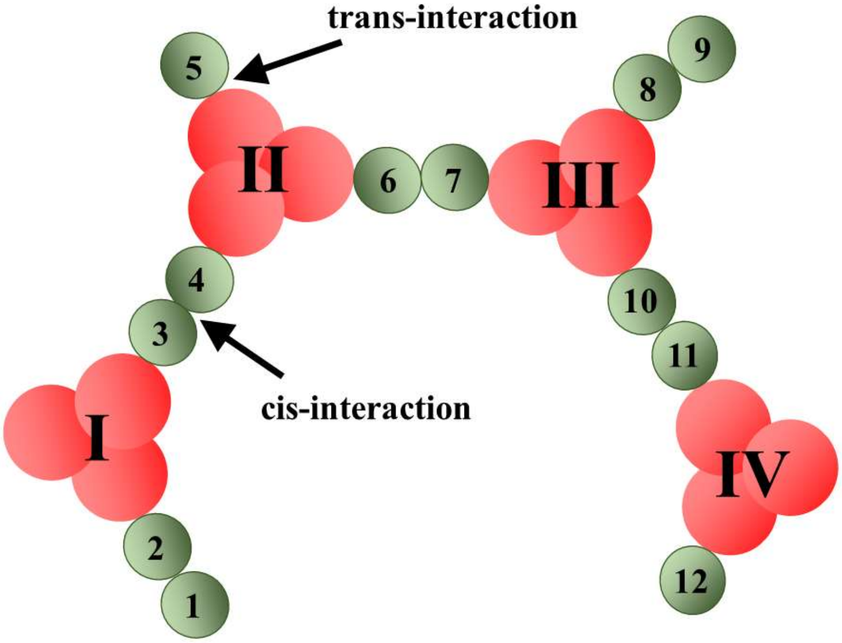

Figure S3: the definition about how to calculate the size of a cluster formed through the combination between ligand-receptor *trans*-interactions and receptor-receptor *cis*-interactions

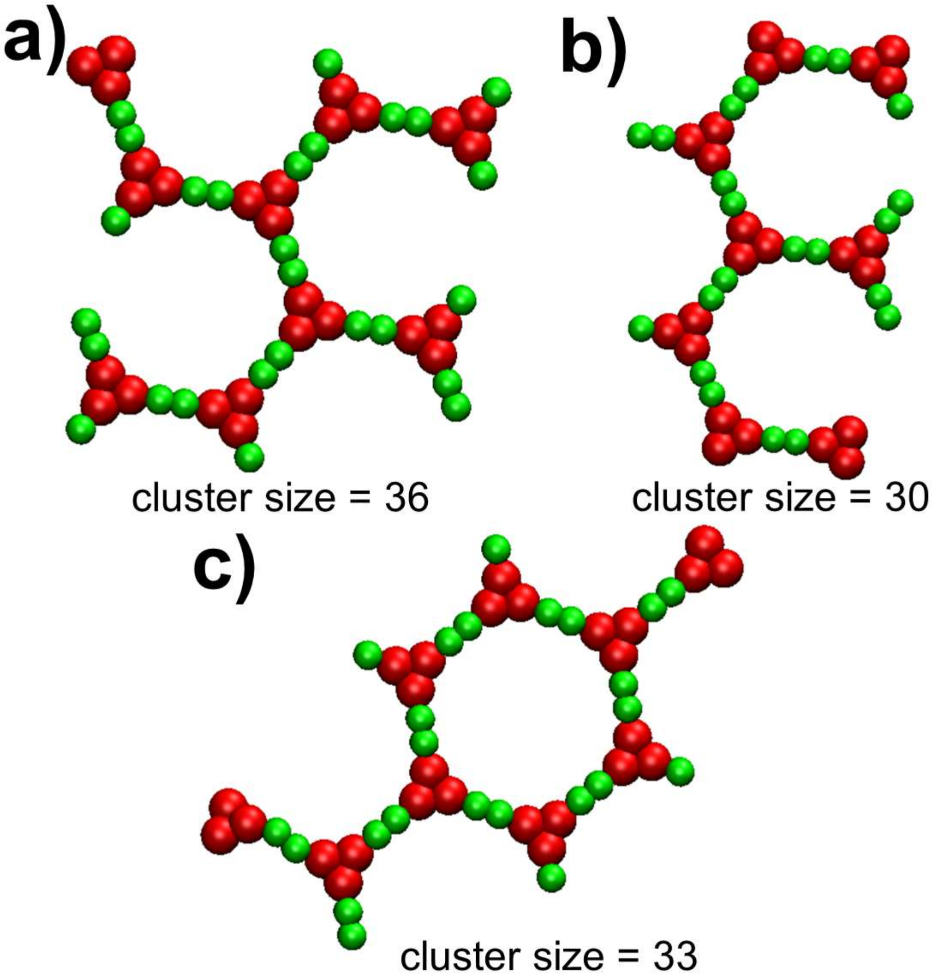

Figure S4: representation of larger clusters selected from different trajectories of sTNFα-TNFR1 system. The size of the first selected cluster equals 36, consisting of 9 ligand trimers and 27 receptors, as shown in **(a)**. The size of the second selected cluster equals 30, consisting of 8 ligand trimers and 22 receptors, as shown in **(b)**. The size of the third selected cluster equals 33, consisting of 9 ligand trimers and 24 receptors, as shown in **(c)**. The figure indicates that hexagonal lattice like structures were formed in all three clusters.
